# Safety and predictors of adverse events during oral immunotherapy with raw egg white

**DOI:** 10.1186/2045-7022-3-S3-O26

**Published:** 2013-07-25

**Authors:** M Vazquez-Ortiz, M Alvaro, O Dominguez, R Jimenez-Feijoo, MT Giner, MA Martin-Mateos, AM Plaza

**Affiliations:** 1 Paediatric Allergy and Clinical Immunology, Hospital Sant Joan de Deu, Barcelona, Spain

## Background

Oral immunotherapy (OIT) for food allergy has shown promising efficacy results. However, safety is still concerning. Objectives. To evaluate safety of egg-OIT. To identify clinical/immunological predictors of adverse events.

## Methods

Prospective longitudinal epidemiological intervention study. Egg-allergic children aged 5–18 underwent a Spanish-approved egg-OIT protocol without premedication.

Clinical data, skin prick test (SPT) and specific IgE (sIgE) at baseline and 9 months after OIT were registered. All dose-related reactions, treatments needed and cofactors involved were recorded. Through survival analysis, we studied the cumulative probability of reactions resolution over time and clinical/immunological risk factors of reactions persistence.

## Results

51 children were recruited. Mean follow-up was 16 months. 74% reached desensitization to one raw egg. 90% of children suffered reactions, 88% of which affected a single organ. Reactions occurred in 7% of doses, being mainly grade 1 (30%) or 2 (32%). Gastrointestinal (37%) and cutaneous (35%) were the most frequent symptoms. Reactions were heterogeneously distributed: (a) 23 children (45%) had occasional symptoms which ceased over time; (b) 28 (65%) children had more frequent and intense symptoms (71% of total reactions). 11 of them, the most difficult cases, had to interrupt the treatment early, whereas in 17 children reactions decreased in frequency but persisted over-time. Reactions persistence was associated with a higher frequency and severity. Kaplan–Meier estimate revealed a cumulative probability of reactions resolution of 25% at 6 months (95% CI: 2.95-9.05) and 50% (95% CI: 7.2-18.8) at 13 months. Cox proportional hazards multivariate regression model identified 2 variables (egg white-sIgE and Sampson’s severity grades 3 or 4 at baseline egg challenge) as independent risk factors of reactions persistence, with hazard ratios of 1.15 (95% CI: 1.05-1.27; p=0.002) and 4.39 (95% CI: 1.78–10.87; p=0.01), respectively. Early withdrawal was associated to pre-existing asthma and higher sIgE levels (p<0.05).

## Conclusion

OIT with raw egg white led to early withdrawal due to adverse events in 21% of children. An additional 33% had persistent reactions over follow-up. Egg white-sIgE, reaction severity at baseline challenge and pre-existing asthma would help clinicians to identify highly reactive patients before egg-OIT. Further research is needed to improve safety before egg-OIT can be extended to routine practice.

## Disclosure of interest

None declared.

